# VPS35 depletion does not impair presynaptic structure and function

**DOI:** 10.1038/s41598-018-20448-4

**Published:** 2018-02-14

**Authors:** Sonia Vazquez-Sanchez, Sander Bobeldijk, Marien P. Dekker, Linda van Keimpema, Jan R. T. van Weering

**Affiliations:** 1 Department of Functional Genomics, Center for Neurogenomics and Cognitive Research, Neuroscience Campus Amsterdam, Vrije Universiteit (VU), Amsterdam, Netherlands; 2 Clinical Genetics, Center for Neurogenomics and Cognitive Research, Neuroscience Campus Amsterdam, VU medical center, Amsterdam, Netherlands; 3 Sylics (Synaptologics BV), PO box 71033, 1008 BA Amsterdam, The Netherlands

## Abstract

The endosomal system is proposed as a mediator of synaptic vesicle recycling, but the molecular recycling mechanism remains largely unknown. Retromer is a key protein complex which mediates endosomal recycling in eukaryotic cells, including neurons. Retromer is important for brain function and mutations in retromer genes are linked to neurodegenerative diseases. In this study, we aimed to determine the role of retromer in presynaptic structure and function. We assessed the role of retromer by knocking down VPS35, the core subunit of retromer, in primary hippocampal mouse neurons. VPS35 depletion led to retromer dysfunction, measured as a decrease in GluA1 at the plasma membrane, and bypassed morphological defects previously described in chronic retromer depletion models. We found that retromer is localized at the mammalian presynaptic terminal. However, VPS35 depletion did not alter the presynaptic ultrastructure, synaptic vesicle release or retrieval. Hence, we conclude that retromer is present in the presynaptic terminal but it is not essential for the synaptic vesicle cycle. Nonetheless, the presynaptic localization of VPS35 suggests that retromer-dependent endosome sorting could take place for other presynaptic cargo.

## Introduction

Retromer is a protein complex that regulates endosomal recycling in all eukaryotic cells^1^. Retromer was first described in yeast^2^ and is highly conserved across all the lineages^1^. The retromer complex is formed by two essential modules: the cargo-selection subcomplex, which binds to the protein that has to be transported, and the membrane deformation subcomplex, which binds to the endosomal membrane to produce the necessary membrane deformation for trafficking (see review^3^). The cargo-selection subcomplex in mammals is constituted by VPS35, VPS29 and VPS26A or VPS26B^4^. VPS35 is the largest protein and the central subunit of this trimetric complex^5^. The membrane deformation subcomplex is constituted of SNX-BAR proteins (SNX1, SNX2, SNX5, SNX6 and SNX32 in mammals). SNX-BAR proteins dimerize in different patterns which leads to a variety of retromer complexes, (although some variants of retromer complex do not contain SNX-BAR proteins, see reviews^6,7^). These two modules together transport the cargo from the endosome to the trans-Golgi network^2^ or to the plasma membrane^8^. Retromer is essential for a great variety of cell functions by specific sorting of membrane proteins: Retromer is involved in Wnt-dependent development^9^, epithelial polarity^10^, neuronal morphogenesis^11–13^, autophagy^14^, nutrition^15^ and lysosomal degradation^16^ among other cell processes. The central role of retromer is also highlighted by the observation that the lack of retromer is lethal during embryonic stages, both in mammalian^17,18^ and fly models^9,10^.

Retromer dysfunction is linked with Parkinson’s and Alzheimer’s disease among other neurological disorders (see review^19^). In fact, increasing retromer stability has been proposed as a therapeutic target for these neurodegenerative diseases^20–22^. Although retromer seems a promising drug target, very little is still known about the neurobiological function of retromer. Hence the physiological role of retromer in the brain needs to be addressed.

The most characteristic neuronal function is to communicate through neurotransmission, a process which takes place at synapses. Functional studies have established that retromer regulates adrenergic and glutamatergic neurotransmitter receptor trafficking to the postsynaptic plasma membrane^12,23,24^. Retromer has been found dynamically localized at the synapses in murine neurons^23,25,26^, and VPS35 is found in synaptosomal membranes and synaptic vesicle enriched fractions isolated from rodent brain^27^. A recent study in *Drosophila* suggests that Vps35 is in presynaptic terminals at the edge of the active zone, where it regulates synaptic vesicle recycling^28^. In both Parkinson’s and Alzheimer’s disease, central proteins that contribute to the pathology are found in presynaptic terminals (APP^29,30^ and α-synuclein^31,32^), which might suggest that retromer-dependent trafficking occurs at presynaptic terminals. However, to our knowledge there is no report investigating retromer role in the mammalian presynaptic terminal.

**Table 1:** Overview of the effect of the different shRNAs against VPS35 in the measured presynaptic variables compared to control. Observed changed are bold. Detailed information (average, SEM, n and statistics) is displayed in Supplementary Table S1.

Figure	measured variable	shVPS35-1	shVPS35-2	shVPS35-3
3C	Dendritic length (µm)	unchanged	unchanged	unchanged
3D	Axonal length (µm)	unchanged	unchanged	unchanged
3F	Synapses/µm (VAMP2)	unchanged	**decreased**	**decreased**
3G	VAMP2 (a.u.)	unchanged	unchanged	unchanged
3H	Synaptophysin-1 (a.u)	unchanged	unchanged	unchanged
3I	Synapses/µm (bassoon)	unchanged	unchanged	unchanged
4B	Active zone length (µm)	unchanged	unchanged	unchanged
4C	# Synaptic vesicles/synapse	unchanged	unchanged	unchanged
4D	# docked synaptic vesicles/synapse	unchanged	unchanged	unchanged
5C	1st peak amplitude (∆F/Fmax)	N.A.	**decreased**	unchanged
5D	% Active synapses	N.A.	unchanged	unchanged
5E	Fmax (a.u.)	N.A.	**decreased**	**decreased**
6C	1st peak amplitude (∆F/Fmax)	unchanged	unchanged	unchanged
6D	% Active synapses	**decreased**	unchanged	unchanged
6E	Fmax (a.u.)	**decreased**	unchanged	unchanged
6F	F Baseline (a.u)	**decreased**	unchanged	unchanged
6G	Ratio peak amplitude (2nd/1st)	unchanged	unchanged	unchanged
6H	F pH = 5.5 (a.u.)	unchanged	unchanged	unchanged

The aim of this study was to determine the role of retromer in presynaptic structure and function. We first investigated the location of VPS35, the core subunit of retromer, in the presynaptic terminal with confocal and immuno-electron microscopy techniques in mouse hippocampal neurons. To address retromer function, we acutely depleted retromer subunit VPS35 to evaluate the impact of this depletion on the presynaptic ultrastructure using immunocytochemistry and electron microscopy, and the impact on synaptic vesicle release and retrieval using life cell imaging with pHluorin secretion reporters (synaptopHluorin^33^ and sypHy^34^).

## Results

### VPS35 is in the presynaptic terminal

**Figure 1:**
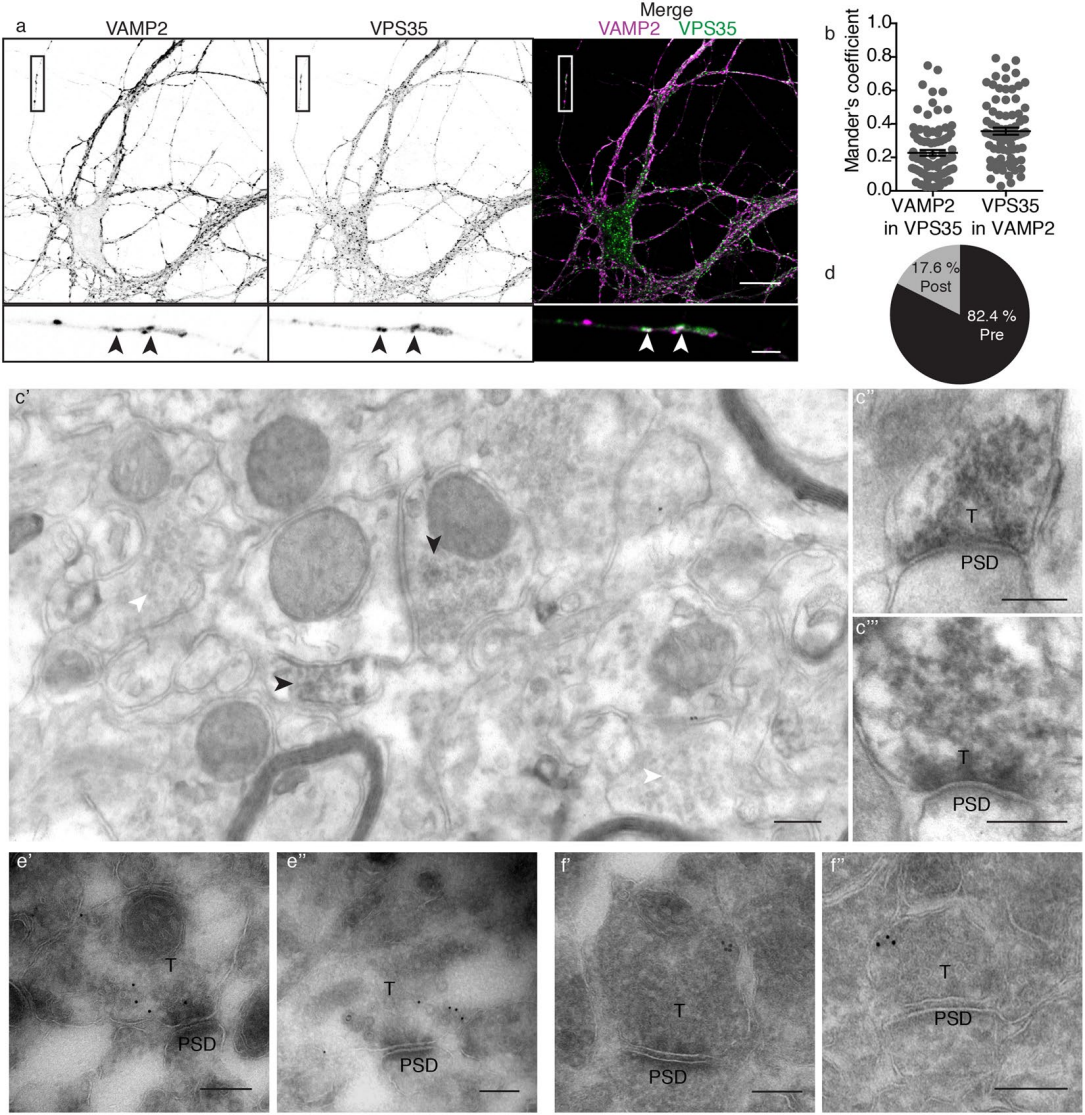
VPS35 is present in presynaptic terminals. (**a**) Representative confocal microscopy images of hippocampal neurons immunolabeled for VAMP2/synaptobrevin and VPS35. Arrowheads indicate co-localization between VAMP2/synaptobrevin and VPS35 puncta. Scale bar of the neuron image = 20 μm, scale bar of the zoomed neurite = 3 μm. (**b**) Mander’s coefficients for the co-localization between VAMP2/synaptobrevin and VPS35 in neurites (n = 78 fields of view, N = 3 animals). (**c**) Representative electron micrographs of hippocampal synapses from 3 independent wild-type mice. Each of the micrographs correspond to a different animal (N = 3 animals). Scale bar = 200 nm. (**c’**) In black arrowheads indicate the synaptic vesicle cloud of DAB positive presynaptic terminals and in white arrowheads DAB negative presynaptic terminals. (**c”**,**c”’**). Zoom in of DAB positive presynaptic terminals. (**d**) Percentage of synapses with VPS35 immunoreactivity in the presynaptic site (82.4% Pre) and postsynaptic site (17.6% Post). (**e’**,**e”**) Immunoelectron micrographs of presynaptic terminals stained with a rabbit antibody against VPS35 labelled with Protein A-10nm gold conjugate. The images are representative of two independent experiments (N = 2 animals). Scale bar = 200 nm (**f’**,**f”**) Immunoelectron micrographs of presynaptic terminals stained with a goat antibody against VPS35 labelled with a secondary antibody rabbit anti-goat and Protein A-10nm gold conjugate. The images are representative of two independent experiments (N = 2 animals). Scale bar = 200 nm. ‘PSD’ indicates postsynaptic side and ‘T’ the presynaptic terminal.

We first characterized the distribution of retromer in mouse hippocampal synapses. We performed immunocytochemistry against VPS35 together with a synaptic marker (VAMP2/synaptobrevin) in cultured wild-type neurons after 14 days *in vitro* (DIV14). We performed co-localization studies only in the neurites in order to exclude the endosomes present in the cell body from the analysis. Approximately 22% of VPS35 immunoreactivity showed also VAMP2/synaptobrevin reactivity (Manders’ coefficient M1: 0.22 ± 0.01), while approximately 35% of synapses contained VPS35 signal (Manders’ coefficient M2: 0.35 ± 0.02) (Fig. 1a,b). Therefore, the confocal data show that retromer can be found at synaptic locations in hippocampal mouse neurites. We immunostained free floating sections of wild-type mouse brain to test in which synaptic compartments VPS35 can be found using electron microscopy. We observed immunoreactivity in hippocampal presynaptic terminals, but not all synapses showed VPS35 immunoreactivity (Fig. 1c’). VPS35-positive presynaptic terminals showed a dark precipitate around the whole synaptic vesicle cloud (Fig. 1c”,c”’). VPS35 immunoreactivity was more frequently found in the presynaptic side (82.4%) than in the postsynaptic side (17.6%; SEM = 1.8, Fig. 1d), and it was not observed in negative controls (blocking peptide, Supplementary Fig. S2). The presynaptic location of VPS35 was verified by immuno-gold electron microscopy using two different antibodies against VPS35 detected by Protein A-gold 10 nm. VPS35 immunosignal of both antibodies was detected inside presynaptic terminals (Fig. 1e–f, Supplementary Fig. S1), but not in the negative controls (absence of primary antibody, Supplementary Fig. S2). VPS35 immuno-gold signal was also present in some postsynaptic structures (Supplementary Fig. S3). Overall, these data show that VPS35 is present in presynaptic terminals, but not all synapses contain retromer.

### Synaptic VPS35 is functionally knocked down by independent shRNAs

**Figure 2:**
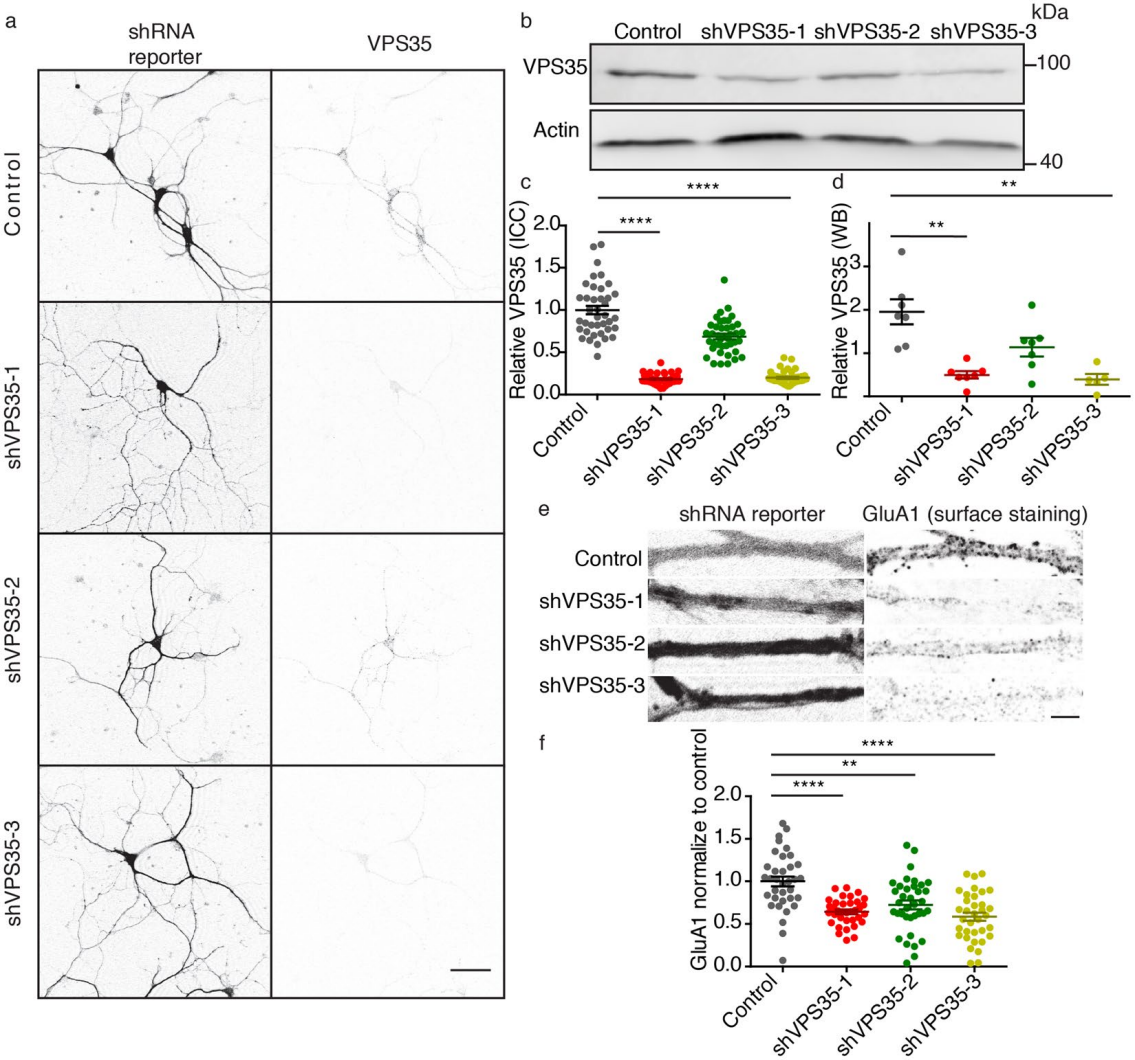
Synaptic VPS35 is functionally knocked down by three independent shRNA. (**a**) Representative confocal microscopy images of cortical neurons infected with control shRNA and the three shRNAs against VPS35. Left, mCherry signal reporting the expression of lentivirus containing the shRNAs coding sequences. Right, neurons immunolabeled for VPS35. Scale bar = 50 μm. (**b**) Representative western blot showing the knock down of VPS35 by three independent shRNAs. Original uncropped blots are shown in Figure S1. (**c**). Quantification of VPS35 intensity in immunostainings (n = 25 ± 1 fields of view, N = 2 animals) (**d**) Quantification of VPS35 levels normalized to total protein levels (assessed by TCE staining) in western blot. Values are presented as a ratio compared to the control condition. (N = 5 ± 1 blots/animals). (**e**) Representative confocal microscopy images of hippocampal neurons expressing either control shRNA or one of the three shRNAs against VPS35. Left, mCherry signal which reports expression of lentivirus containing the shRNAs. Right, surface immunolabelling of GluA1. Scale Bar = 5 μm. (**f**) Quantitative analyses of GluA1 staining intensity (n = 35 ± 1 fields of view, N = 3 animals). Detailed information (average, SEM, n and statistics) is shown in Supplementary Table S1.

VPS35 is a crucial protein for maintaining the stability of all known retromer complexes^20,35^. In order to decrease retromer levels and to be able to define the role of retromer at the presynaptic terminal, we designed three short hairpin RNAs (shRNA) against VPS35. Previous studies documented that retromer depletion affects hippocampal development inducing defects in neurites and spine density^12,13^; hence we aimed to bypass possible changes in neuronal morphology, which may confound our presynaptic functional studies. Therefore, we infected cultured neurons with lentivirus particles containing shRNAs when the neurons already formed synapses (DIV7). We assessed the shRNA efficiency in reducing VPS35 protein in neurons at DIV14-DIV15 by quantifying VPS35 protein levels using both immunocytochemistry and Western blot. The immunostaining of primary cortical neuron network cultures revealed that the three shRNA were able to reduce VPS35 expression: shVPS35-1 significantly reduced the VPS35 staining by 81%, shVPS35-3 significantly reduced VPS35 by 80%, while shVPS35-2 reduced the VPS35 signal just by 31%, which was not significantly different compared to control levels (Fig. 2a,c; Supplementary Table S1). These results were replicated by Western Blot (Fig. 2b,d). Therefore, we conclude that VPS35 protein level was reduced by independent shRNAs.

Next, we tested if the VPS35 depletion induced a functional impairment in retromer in neurons. Previous studies showed that impairment in retromer-SNX27 pathway results in a reduction of GluA1 at the neuronal surface^12,24^. In accordance to these studies, we find that the three shRNA against VPS35 lead to a significant reduction of GluA1 surface staining compared to control (GluA1 levels significantly reduced by 35% (shVPS35-1), 27% (shVPS35-2) and 41% (shVPS35-3) (Fig. 2e,f; Supplementary Table S1). We conclude that seven-day infection with all three independent shRNAs against VPS35 led to retromer dysfunction in mouse neurons.

### Presynaptic structure is not affected by knocking down VPS35 in neurons

**Figure 3:**
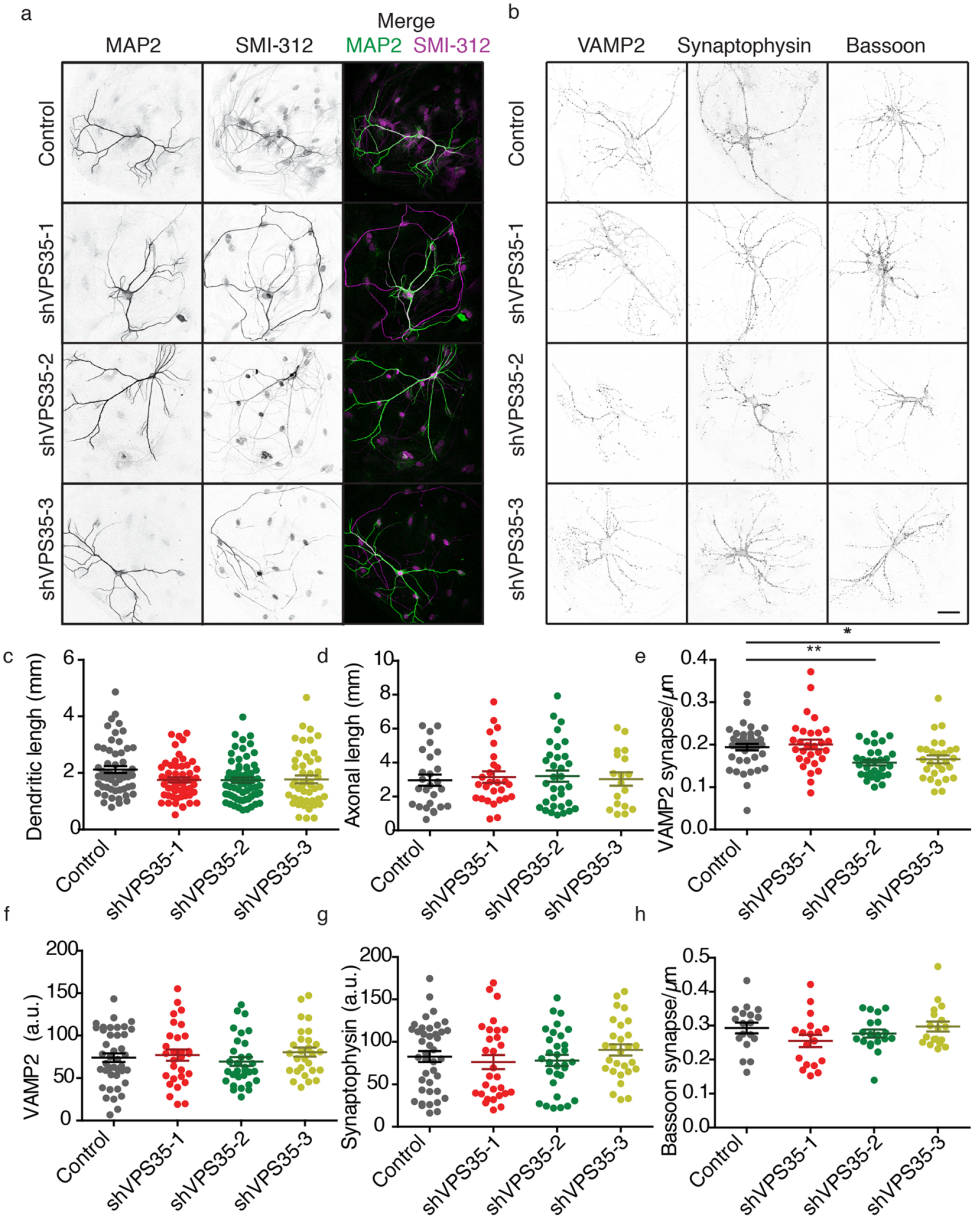
Neuronal morphology is not affected by knocking down VPS35 in neurons. (**a**,**b**) Representative confocal microscopy images of hippocampal autaptic neurons containing either control shRNA or one of the three shRNAs against VPS35. Scale bar = 50 μm. (**a**) Immunolabelling for MAP2 and SMI-312. Merge image of the MAP2 (green) SMI-312 (magenta). (**b**) Immunolabelling for VAMP2/synaptobrevin, Synaptophysin and Bassoon. Quantification of (**c**) the dendritic length (n = 56 ± 8 neurons, N = 5 animals); (**d**) axonal length n = 38 ± 8, N = 3 animals); (**e**) VAMP2/synaptobrevin-labelled synaptic density (n = 34 ± 7 neurons, N = 3 animals); (**f**) of VAMP2/synaptobrevin staining intensity n = 34 ± 7 neurons, N = 3 animals). (**g**) synaptophysin staining intensity (n = 34 ± 7 neurons, N = 3 animals). (**f**) Bassoon staining intensity (n = 20 ± 1 neurons, N = 2 animals). Detailed information (average, SEM, n and statistics) is shown in Supplementary Table S1.

To test if the acute retromer depletion bypasses the previously reported morphological defects^12,13^, we stained autaptic neurons at DIV14-15 for dendritic (MAP2), axonal (SMI-312) and synaptic (VAMP2/synaptobrevin, synaptophysin and bassoon) markers. We used autaptic neuronal cultures as they allowed us to measure the neuritic arbor from a single neuron^34^. We analyzed the confocal images using SynD, a semi-automated image analysis routine^36^. The overall neuron morphology did not change upon VPS35 knock down (Fig. 3a,b). The following morphological parameters did not differ between the control and VPS35 shRNA-expressing neurons: dendritic length, axonal length, synapse density calculated as bassoon positive puncta per µm of neurite and, the expression levels of synaptic vesicle proteins such as VAMP2/synaptobrevin and Synaptophysin (Fig. 3c,d,f–h; Supplementary Table S1). We observed a significant difference in the number of VAMP2/synaptobrevin positive puncta per µm, which was reduced by ∼18% in shVPS35-2 and shVPS35-3 infected neurons compared to control (Fig. 3e; Supplementary Table S1). To verify that changes in neurite length can be detected in this assay, we compared an early time point (DIV4) of autaptic neuronal cultures, when neuron morphology is less complex, with the mature time point used in this study (DIV14). Quantitative analysis detected both axonal and dendritic length increased significantly during neuronal development (Supplementary Fig. S5). In addition, this methodology has been used in our laboratory previously^37–40^. Together, these data show that acute knock down of VPS35 in mature neurons does not alter most aspects of neuronal morphology.

**Figure 4:**
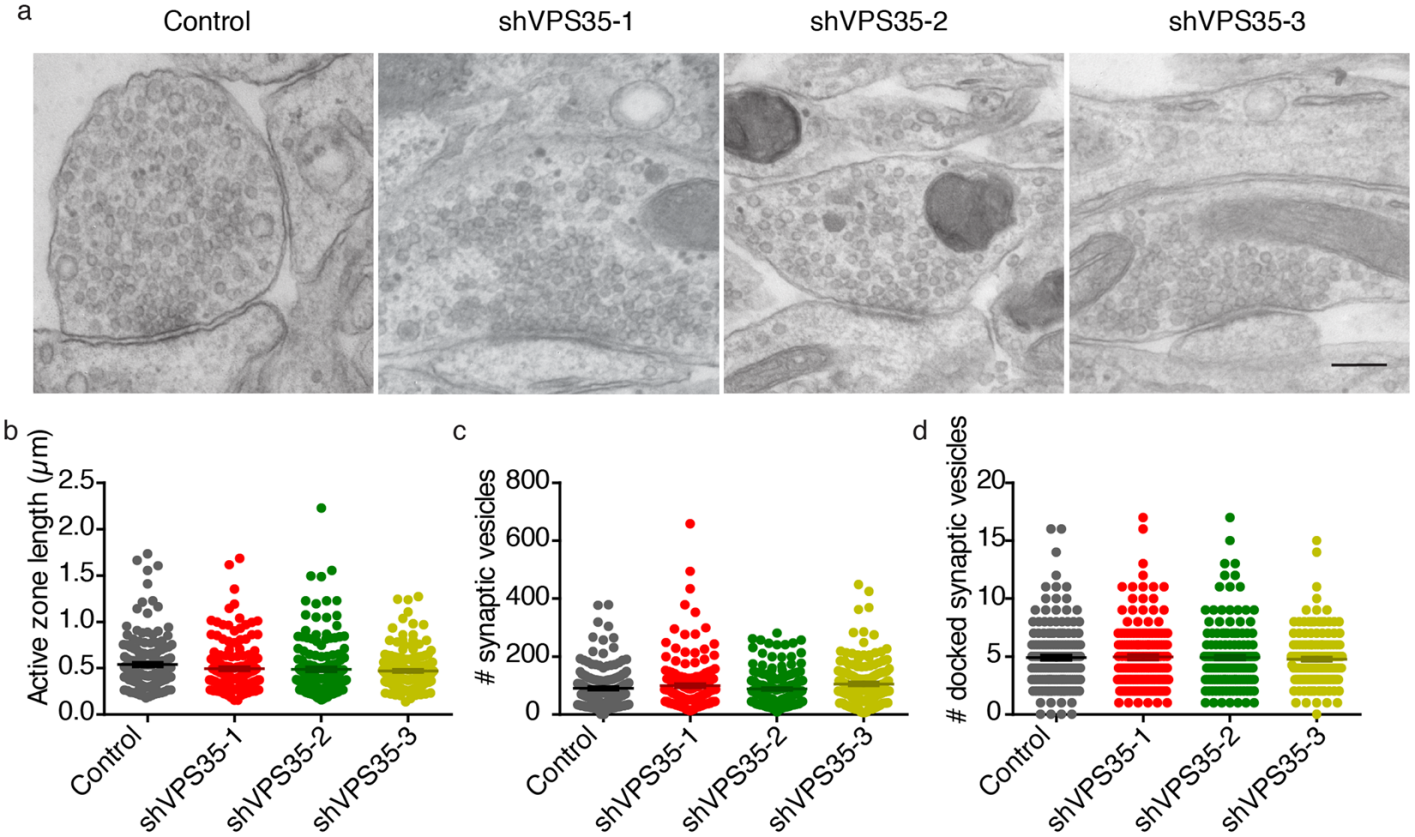
Presynaptic nanostructure is not altered in VPS35 KD neurons. (**a**) Typical examples of electron micrographs of hippocampal synapses from control and VPS35 KD neurons. Scale bar = 200 nm. The quantitative parameters (**b**) active zone length (**c**) total number of synaptic vesicles and (**d**) docked synaptic vesicles are indicated as bar graphs. (n = 162 ± 3 synapses, N = 3 animals). Detailed information (average, SEM, n and statistics) is shown in Supplementary Table S1.

We next explored the presynaptic ultrastructure upon VPS35 knock down by Transmission Electron Microscopy (TEM) using aldehyde fixation at DIV14–15. The overall synaptic morphology of shRNA-expressing neurons did not show abnormalities (Fig. 4a). The active zone length, the total amount of synaptic vesicle and, the number of docked synaptic vesicles did not differ between the four groups (Fig. 4b–d). These data show that acute VPS35 depletion does not affect presynaptic ultrastructure.

### Synaptic vesicle release is not altered by knocking down VPS35

**Figure 5:**
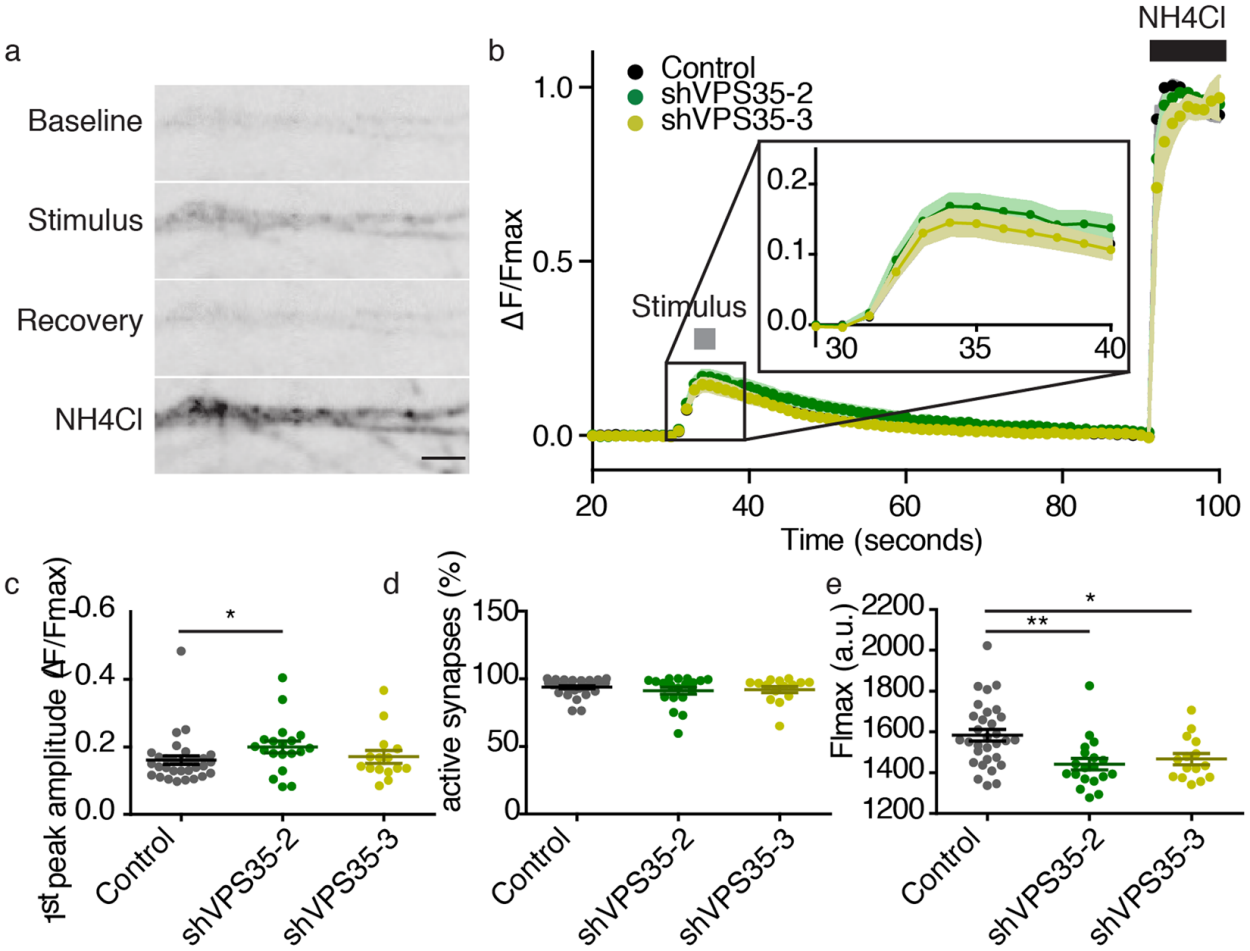
VPS35 KD does not affect SypHy release or retrieval. (**a**) Representative sypHy fluorescence images of neurites during baseline, stimulation, the recovery period and the exposure to NH_4_Cl. Scale bar = 10 µm. (**b**) Time course of sypHy fluorescence during the imaging protocol, plotted as ΔF/Fmax. The grey box indicates the electrical stimulation (100 AP, 40 Hz, 30 mV) and the black box the exposure to 10 seconds of NH_4_Cl (n = 23 ± 8 fields of view, N = 4 animals). (**c**) Maximum response amplitude during the electrical stimulation plotted as ΔF/Fmax. (**d**) Percentage of responsive synapses during the stimulation. (**e**) Maximum response to the exposure to NH_4_Cl. Detailed information (average, SEM, n and statistics) is shown in Supplementary Table S1.

We tested the potential role of VPS35 in presynaptic function by using fluorescent reporters of synaptic vesicle release and retrieval. First, we used sypHy, a pH-sensitive variant of GFP fused in the luminal domain of the synaptic vesicle protein synaptophysin-1^33^. This pH-sensitive reporter allows the visualization of both synaptic vesicle release and retrieval. Our protocol consisted of an electrical stimulation (100 AP, 40 Hz, 30 mA) to evoke synaptic vesicle release followed by an exposure to NH_4_Cl (de-quenching all sypHy) to quantify the total reporter pool (Fig. 5a,b). The reporter showed a punctate pattern upon NH_4_Cl application which is used to place regions of interest (ROIs) for sypHy measurements (Fig. 5a). In this experiment, we excluded neurons infected with shVPS35-1 because they failed to show a punctate sypHy pattern upon NH_4_Cl exposure, which is required for data analysis (data not shown). Control, shVPS35-2 and shVPS35-3 expressing neurons showed a similar time course of fluorescence intensity with a timed response to the electrical stimulation and recovery back to baseline fluorescence within 60 seconds after the stimulus (Fig. 5b). The synaptic vesicle release, which is measured as peak amplitude, was not significantly different between the control neurons and neurons expressing shVPS35-3, but it was increased compared with shVPS35–2 (Fig. 5c). The three groups showed the same percentage of active synapses defined as the percentage of ROIs that respond both to electrical stimulation and NH_4_Cl perfusion (Fig. 5d). The total pool of sypHy was significantly reduced in VPS35-depleted neurons compared with control (Fig. 5e; Supplementary Table S1). These data suggest that VPS35 KD does not affect synaptic vesicle release or retrieval.

**Figure 6:**
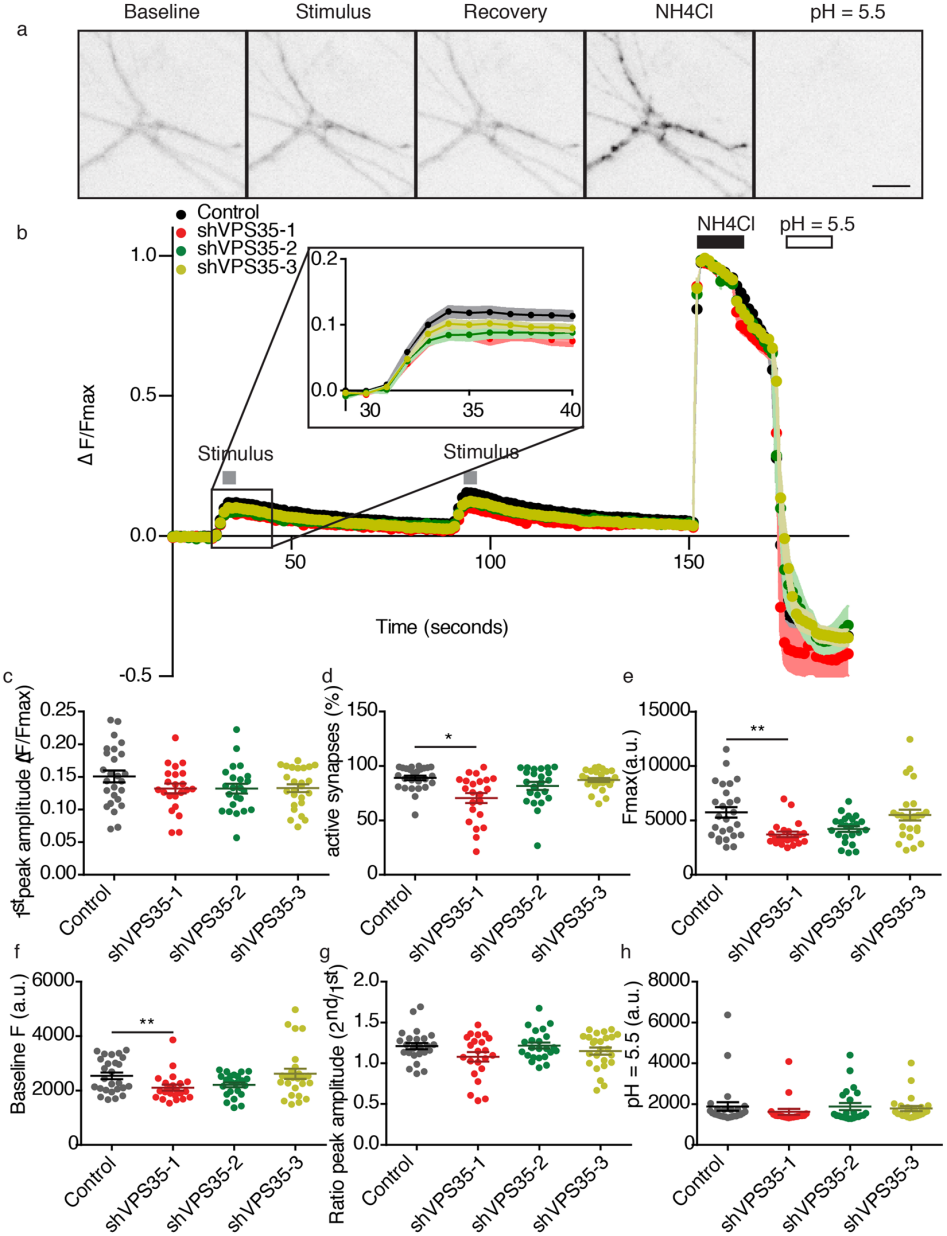
Repetitive stimulation does not induce presynaptic failure in VPS35 KD neurons. (**a**) Representative synaptopHluorin fluorescence images of neurites during the base line, the first stimulation, the first recovery period, the exposure to NH_4_Cl and the exposure to pH = 5.5. Scale bar = 40 µm. (**b**) Time course of synaptopHluorin fluorescence during the imaging protocol, plotted as ΔF/Fmax. The grey boxes indicate the electrical stimulation (100 AP, 40 Hz, 30 mV each), the black box the duration of the exposure to NH_4_Cl and the white box the duration of the exposure to pH = 5.5. (n = 24 ± 2 fields of view, N = 3 animals). (**c**) Maximum response amplitude during the electrical stimulation plotted as ΔF/Fmax. (**d**) Percentage of responsive synapses during the stimulation. (**e**) Maximum synaptopHluorin levels during exposure to NH_4_Cl. (**f**) Average fluorescence of synaptopHluorin during baseline recordings. (**g**) Ratio of the maximum synaptopHluorin fluorescence amplitude between the first and the second electrical stimulation. (**h**) Minimum response to the exposure to pH = 5.5. Detailed information (average, SEM, n and statistics) is available in Supplementary Table S1.

As the effect of shVPS35–1 could not be evaluated using sypHy, we tested also synaptopHluorin as an alternative reporter of synaptic vesicle release and retrieval. SynaptopHluorin is a pH-sensitive variant of GFP fused to the luminal domain of VAMP2/synaptobrevin which works as sypHy, but it shows more cell surface expression which increases the background fluorescence^34^. Hence, at the end of the protocol using this reporter, we exposed the neurons to a pH ~ 5.5 solution to calculate the fraction of synaptopHluorin that remained in the plasma membrane. We also added a second stimulation to the new protocol (identical to the first one) to measure if retromer depleted neurons were able to efficiently release synaptic vesicles after having been already electrically stimulated, which would indicate if retromer is involved in refilling the releasable synaptic vesicles pools. Using SynaptopHluorin as reporter, all the experimental groups showed the typical puncta pattern when treated with NH_4_Cl (Fig. 6a); thus, all groups were included in analysis. All neurons showed a similar time course of fluorescence intensity during the protocol and peak amplitude to the first response (Fig. 6b,c). Compared to control, shVPS35–2 and shVPS35–3 expressing neurons showed the same number of active synapses and the same baseline fluorescence. However, shVPS35–1 infected neurons showed a significant decrease in these two parameters compared to control (Fig. 6d,f; Supplementary Table S1). Neurons infected with shVPS35–3 showed the same response to NH_4_Cl, but shVPS35–1 and shVPS35-2 infected neurons showed a decreased response compared to control (Fig. 6e). The ratio between the fluorescence peaks after stimulation was equal between all the groups (Fig. 6g). The fluorescence during the pH 5.5 wash was also similar between all experimental groups (Fig. 6h). To ensure that the life cell imaging methodology was working as described in literature^33,34^ and as previously in our laboratory^37–39,41^, we assessed the effect of VPS35 depletion in presence and absence of calcium (Supplementary Fig. S6). When calcium is present, both control and the VPS35 knock down showed a similar amount of synaptic vesicle release, but when calcium is absent, synaptic vesicle release is significantly decreased in both groups. These results show that the assay can register changes in synaptic vesicle release. Together, the experiments performed with sypHy or synaptopHluorin show that acute VPS35 depletion does not affect synaptic vesicle release and retrieval, suggesting that retromer does not affect the synaptic vesicle cycle.

## Discussion

The present study addressed the effect of retromer dysfunction in presynaptic structure, and synaptic vesicle release and retrieval. To avoid the potential interference of VPS35 depletion during development, we acutely knocked down VPS35 in neurons after synapse formation. Acute VPS35 depletion resulted in retromer dysfunction, which was measure as a decrease in GluA1 receptors in the plasma membrane^12,24^. Our results show for the first time that retromer is present at the mammalian presynaptic terminal. VPS35 depletion did not affect most measured neuronal features: neuronal morphology (neurite length and synapse number), presynaptic ultrastructure, and synaptic vesicle release and retrieval (Table 1). The data show that presynaptic retromer is not essential for basic presynaptic structure and function.

To determine the role of presynaptic retromer we have used a shRNA approach. shRNAs are widely used to acutely deplete proteins, but this method is susceptible to off target effects (see reviews^42,43^). The off-target effects are those genes or processes which are affected by the shRNA that are not the target, in this case VPS35. The three shRNAs have been validated to functionally inhibit retromer by impairing GluA1 surface localization, which has been described to be retromer-dependent by several laboratories^12,24^. To avoid the phenotypic association with off-target effects, only phenotypes that are replicated by all three shRNAs against VPS35 are considered to be VPS35-dependent processes in this study.

Several studies have shown that chronically decreasing retromer levels causes defects in neuronal morphology and synapse formation^12,13,44^. We aimed to acutely induce retromer dysfunction in maturing neurons to circumvent these neuronal morphology defects, which might interfere in the evaluation of retromer role in the presynaptic terminal presynaptic terminal. We did not find changes in any of the measured parameters: dendritic length, axonal length, and the number of synapses or synaptic proteins, avoiding the interference of retromer dysfunction during the initial stages of neuronal network development. We did observe a small reduction of ∼18% in the number of VAMP2/synaptobrevin positive puncta per µm of neurite (Fig. 3e) in shVPS35-2 and shVPS35-3, which might suggest that the number of synapses is reduced. However, this reduction was not observed for synapse marker bassoon (Fig. 3h). In addition, shVPS35-1 conditions did not replicate this effect on VAMP2/synaptobrevin puncta (Fig. 3f). Together these results suggest that the reduction in VAMP2/synaptobrevin puncta does not represent a significant loss of synapses. Hence, we conclude that retromer is not required for maintenance of existing synapses and formation of new synapses in mature neurons.

Retromer depletion did not affect presynaptic ultrastructure. Tian *et al*.^12^ reported an increase in synaptic vesicles in VPS35 haploinsufficient presynaptic terminals. This increase in synaptic vesicles was coupled with a decrease in neurotransmitter receptor at the postsynaptic sites. Hence, the alteration in presynaptic ultrastructure was interpreted as a compensatory mechanism to the impairment in the postsynapse. We observed the decrease in glutamatergic receptor labeling at the cell surface, but the number of synaptic vesicles was not altered, suggesting that such potential secondary effects were indeed circumvented by transient retromer depletion (Figs 2e,f and 3a,c). In *Drosophila*, retromer depletion led to a decrease in the number of synaptic vesicles, which was interpreted as a defect in endocytosis and regeneration of the synaptic vesicles due to the lack of retromer^28^. Previous work of this laboratory has shown that addressing presynaptic ultrastructure with TEM is sensitive to detect changes^40,45–49^. However, upon VPS35 knock down we did not observe such an effect. The differences between literature and our data suggest that retromer function may be different in different organisms, or in different synapses, and/or it may change during developmental stages.

Retromer depletion did not affect synaptic vesicle release and retrieval as measured by pH-sensitive reporters. In contrast, VPS35 knock down reduced the total expression of these reporters compared to control (except for shVPS35-3 in SynaptopHluorin experiments) (Figs 5e and 6e). In the shVPS35-1 condition, the sypHy reporter even failed to show a punctate synaptic localization. The reduced expression of these reporters was not mirrored by the endogenous proteins (VAMP2/synaptobrevin or Synaptophysin; Fig. 3f,g; Supplementary Table S1), or a general reduction in synaptic vesicles (Fig. 4c). These observations might suggest that retromer is involved in the targeting of newly or exogenous expressed proteins to synaptic terminals. Experiments using labelling of endogenous *de novo* synthesized proteins may shed light on this. However, the inconsistencies of the observations between groups suggest that the reduction in fluorescent reporter expression could be mediated by non-specific off-target effects of the shRNA approach.

Acute VPS35 depletion does not affect the presynaptic structure and synaptic vesicle release; however, retromer is present in presynaptic terminals. We used immuno-electron microscopy against VPS35 to address VPS35 localization in mammalian synapses for the first time. The nano-resolution of this technique allowed us to demonstrate that VPS35 is present in the murine hippocampal presynaptic terminal, which is in line with a recent study that showed that Vps35 is in the *Drosophila* presynaptic terminal^28^. A main question remains unanswered: why retromer is present in mammalian presynaptic terminals but does not affect the dominant membrane recycling pathway in that region, the synaptic vesicle cycle. Our confocal data show that about 35% of hippocampal synapses contain retromer, suggesting that retromer may play a role in just a subset of synapses. We hypothesized that retromer plays a role in the modulation of presynaptic communication. Recently, it has been shown that dopamine transporter availability at the cell surface is regulated by retromer^50^. Dopamine transporter mediates the presynaptic reuptake of dopamine, which determines dopaminergic neurotransmission. Potentially retromer may also recycle neurotransmitter transporters in hippocampal terminals. Retromer also mediates G protein–coupled receptors (GPCRs) signaling (see review^51^). GPCRs can be localized at presynaptic terminals and are important regulators of synaptic communication (see review^52^). Hence, through these two recycling pathways, which would not directly affect parameters tested in this study, retromer might be involved in the modulation presynaptic communication.

Our goal was to investigate the role of retromer in presynaptic terminals in terms of structure and synaptic vesicle release. We found that retromer is present at the mammalian presynaptic terminal. However, acute retromer depletion did not affect any of the measured structural and functional parameters. The fact that retromer is present the presynaptic terminals could be further investigated to elucidate the role of retromer in recycling presynaptic membrane proteins such as neurotransmitter transporters or GPCRs.

## Materials and Methods

### Plasmids

The target sequences of the shRNAs were as follows: CGT GTG GAC TAC GTC GAT AAA (shVPS35-1), CCA AAT CTT GAG TCC AGT GAA (shVPS35-2), GCT GTC ACC AAA GAG TTA CTA (shVPS35-3), TTC TCC GAA CGT GTC ACG T (shControl, scramble^53^). The target sequences were cloned in to a lentiviral expression vector under the U6 promotor containing mCherry under synapsin promotor, which was used as a reporter of the lentiviral infection.

To report synaptic vesicle release we used synaptophysin-pHluorin under synapsin promotor (sypHy)^33^ and synaptobrevin-pHluorin under synapsin promotor (synaptopHluorin)^34^.

### Laboratory animals

Animal experiments were approved by the animal ethical committee of the VU University/VU University Medical Centre (“Dier ethische commissie (DEC)”; license number: FGA 11-03) and, they are in according to institutional and Dutch governmental guidelines and regulations.

### Primary cell culture

Mouse E18 hippocampi or cortices were dissected in Hanks balance salt solution (HBSS, Sigma) with 10 mM HEPES (Life Technologies) and digested by 0.25% trypsin (20 minutes at 37 °C; Life technologies) in HBSS. The tissue disassociation was performed with fire-polished Pasteur pipettes in DMEM with FCS. The neurons were spun down and re-suspended in neurobasal medium with 2% B-27, 18 mM HEPES, 0.25% glutamax and 0.1% Pen-Strep (Life Technologies). For VPS35 protein quantification (Western Blot), 150,000/mL cortical neurons were plated in coated plates, and for immunocytochemistry 50,000/mL hippocampal neurons on coated coverslips with poly-L-ornithine (PLO, Sigma) and laminin (Sigma). For morphological characterization, 1,300/mL hippocampal neurons were plated on astrocyte micro-islands^48^. For electron microscopy and life cell imaging 25,000/mL hippocampal neurons were plated in a monolayer of astrocytes. Neurons were maintained at 37 °C and 5% CO_2_ until the day of the experiment.

### Western Blot

Neurons at DIV14–15 were washed with ice-cold phosphate-buffered saline (PBS), scraped and lysed in loading buffer. Samples (300.000 neurons each) were boiled for 10 minutes at 90 °C, run in SDS-PAGE (10% 1 mm acrylamide gel with 2, 2, 2-Trichloroethanol) and, transferred into Polyvinylideenfluoride (PVDF) membranes (Bio-rad) (1 hour, 0.3 mA, 4 C). Membranes were blocked and incubated with primary antibodies (2 hours, room temperature) in PBS-T and 5%milk (VPS35 1:500, Abcam, Cat. No. ab10099), (actin 1:10000, Chemicon, Cat. No. MAB1501) incubated with secondary alkaline phosphatase conjugated antibodies (1:10000, Sigma) in PBS-T and 5% milk (1 hour, room temperature), incubated 5 minutes with AttoPhos (Promega) and, scanned with a FLA-5000 fluorescent image analyzer (Fujifilm).

### Immunocytochemistry and Confocal Imaging

Neurons at DIV 14–15 were fixed in 4% paraformaldehyde in PBS, permeabilized with 0.5% Triton X-100 and, blocked with 2% normal goat serum and 0.1% Triton X-100 in PBS. The primary antibodies used were MAP2 (1:20000, Abcam, Cat. No. ab5392), SMI-312 (1:5000, Abcam, Cat. No. ab24574), VAMP2/synaptobrevin (1:1000, Synaptic Systems, Cat. No. 104 211), Bassoon (1:500, Enzo Life Science, Cat. No. SAP7F407), Synaptophysin-1 (1:1000, SynapticSystems, Cat. No. 1011004), VPS35 (1:500, Abcam, Cat. No. ab97545), GluA1 (1:50, Merk Millipore, Cat. No. MAB2263). The secondary antibodies were conjugated to Alexa dyes (1:1000, Molecular Probes). The cells were mounted on microscope slides with Dabco-Mowiol (Invitrogen). Image acquisition was performed on a Carl Zeiss LSM510 confocal microscope, with a Plan-Neofluar 40×/1.3 oil objective. Colocalization analysis was performed using JACoB plugin in zoomed neurites^54^. Morphological analysis was performed using SynD^36^.

### Electron microscopy

For pre-embedding immunolabelling of VPS35, whole wild-type mouse brains were immersion-fixed in 4% paraformaldehyde in 0.1 M phosphate buffer (PB, pH 7.4), cryo-protected in 30% sucrose and frozen at −80 °C. Endogenous peroxidase in 40 μm cryosections was quenched by 0.3% H_2_O_2_ and 10% methanol in PBS. The sections were treated with 1 freeze–thaw cycle and blocked with 0.1% BSA in PBS. The primary anti-VPS35 antibody (1:250, Abcam, Cat. No. ab10099) labelled free-floating sections for 1 h at room temperature and was detected by a biotinylated rabbit anti-goat antibody (Jackson ImmunoResearch, Cat. No. 305065003), avidin–biotin horseradish peroxidase complex formation (VECTASTAIN ABC kit; Vector Laboratories, Burlingame, CA), and 3′-3′-diaminobenzidine (DAB) precipitation (DAB Substrate Kit, Vector Laboratories). As a negative control, primary antibody was preincubated with blocking peptide (Abcam, Cat. No. ab23181 at a ratio of 5:1) for 30 minutes at room temperature with agitation. The sections were contrasted by 1% osmium tetroxide and 1.5% potassium ferricyanide, dehydrated though increasing ethanol concentrations (30%, 50%, 70%, 90%, 96%, 100%), and embedded in epoxy resin. Hippocampal regions were cut into 80 nm sections for transmission electron microscopy (TEM) analysis in a JEOL1010 electron microscope (JEOL, Tokyo, Japan). Digital images of regions with immunoreactivity were acquired by a side-mounted CCD camera (Morada; Olympus Soft Imaging Solutions, Münster, Germany) and iTEM analysis software (Olympus Soft Imaging Solutions). Two independent researchers counted the presence of immunoreactivity in the presynaptic or postsynaptic side to calculate the percentages of Fig. 1d.

For ultrastructural characterization of VPS35 KDs, neurons at DIV14–15 were fixed and flat embedded. Cells were fixed for 1 hour with 2.5% glutaraldehyde (GA, Merck) in 0.1 M cacodylate buffer, pH 7.4, after cell were wash and stained 1 hour at room temperature with 1% OsO_4_/1% KRu(CN)_6_ in milliQ water. Then cells were embedded in epoxy resin and sectioned as described above. Cells were stained using in uranyl acetate and lead citrate in Ultra stainer LEICA EM AC20. Images were acquired at 60.000 × magnification using the TEM set-up described above.

For immuno-gold TEM, hippocampi of 2 months mice were fixed in 4% PFA with 0.1% GA in 0.1 M PB and embedded in increasing concentrations of gelatin at 37 °C. The hippocampi were infiltrated in 2.3 M sucrose at 4 °C and frozen in liquid nitrogen. Seventy nm thick sections were obtained with a cryo-ultramicrotome (UC6, Leica), collected at −120 °C in 1% methyl-cellulose in 1.2 M sucrose and transferred onto formvar/carbon-coated copper mesh grids. The sections were washed with PBS at 37 °C treated with 0.1% glycine, and immunolabelled. VPS35 (1:200, Abcam, Cat. No. ab97545) was diluted in PBS with 0.1% BSA and VPS35 (1:200, Abcam, Cat. No. ab10099) was diluted in PBS with 0.1% of BSA and 0.1% cold water fish gelatin and detected by a rabbit anti-goat antibody (1:200, Jackson ImmunoResearch, Cat. No. 305065003). The antibodies were detected with Protein A-10 nm gold (CMC, UMC Utrecht, Netherlands). The negative controls were processed in parallel without primary antibody. The sections were counterstained with 0.4% uranyl acetate in 1.8% methyl-cellulose on ice and imaged on a Tecnai 12 Biotwin transmission electron microscope (FEI Company).

### Live cell Imaging

Neurons at DIV 14–15 were placed in the imaging chamber containing Tyrode’s solution (2 mM CaCl_2_, 2.5 mM KCl, 119 mM NaCl, 2 mM MgCl_2_, 30 mM glucose, 25 mM HEPES, 50 μM AP5 and 10 μM DNQX at pH 7.4). The experiment was performed at room temperature with perfusion of 1 ml per minute of Tyrodes buffer. Images were acquired with the Axiovert II microscope (Zeiss, Oberkochen, Germany) with a 40× oil objective (NA 1.3). The filters were 488 ± 5 nm (emission) and 525 ± 25 nm (excitation) for pHluorin, and 514 ± 5 nm (emission) and 625 ± 27,5 nm (excitation) for mCherry as shRNA reporter. The imaging protocols included 30 first seconds of base line recording, one or two identical stimulation (2,5 seconds at 40 Hz and 30 mA) followed by one minute of recovery time and a final 10 seconds perfusion of NH_4_ (2 mM CaCl_2_, 2.5 mM KCl, 119 mM NaCl, 2 mM MgCl_2_, 30 mM glucose, 25 mM HEPES, 50 mN NH_4_Cl at pH 7.4). As specified in the result section also a final 10 seconds acid perfusion during was applied (2 mM CaCl_2_, 2.5 mM KCl, 119 mM NaCl, 2 mM MgCl_2_, 30 mM glucose, 25 mM MES at pH 5.5). Fluorescence puncta during NH4 exposure (synaptic locations) were analyzed as regions of interest of 4 by 4 pixels’ radium (ROIs). Fluorescence during depolarization of neurons was normalized to baseline and the maximum fluorescence during NH_4_Cl perfusion. The results for each ROI were averaged for each field of view and presented as data points. Fields of view were excluded if a technical problem was detected that could disturb the results.

### Statistical Analysis

Data are expressed as mean values ± standard error of the mean (SEM). The Shapiro-Wilk normality test was used to evaluate the distribution of the data. Bartlett’s test was used to test homoscedasticity. In case data were normally distributed and homoscedastic, data were compared by one-way analysis of variance (ANOVA). Dunnets post-hoc tests were performed after a significant effect was detected by comparing the different knock down groups to the control. When data were not normality distributed and homoscedastic, the Kruskal-Wallis test was used with Dunn’s multiple test as post-hoc. When P-values were lower than 0.05, significance was noted in the figure as: *P < 0.05, **P < 0.01, ***P < 0.001, ****P < 0.0001. Detailed information (average, SEM, n and statistics) is shown in Supplementary Table S1.

### Data availability

The datasets generated and analyzed during the current study are available from the corresponding author on request.

## Electronic supplementary material

Supplementary information
