# Supplementary material for: VPS35 depletion does not impair presynaptic structure and function
Source: Sci Rep. 2018 Feb 14;8:2996. doi: 10.1038/s41598-018-20448-4 (PMC5812998; doi:10.1038/s41598-018-20448-4)
Supplement: Supplementary file 1 — Supplementary information [file 41598_2018_20448_MOESM1_ESM.pdf]

Supplementary files

VPS35 depletion does not impair presynaptic structure and function

Sonia Vazquez-Sanchez[1], Sander Bobeldijk[1], Marien P. Dekker[2], Linda van Keimpema[1,3], and Jan R.T. van Weering [2,*]

[1]Department of Functional Genomics, Center for Neurogenomics and Cognitive Research, Neuroscience Campus Amsterdam, Vrije Universiteit (VU), Amsterdam, Netherlands
[2]Clinical Genetics, Center for Neurogenomics and Cognitive Research, Neuroscience Campus Amsterdam, VU medical center, Amsterdam, Netherlands
[3]Sylics (Synaptologics BV), PO box 71033, 1008 BA, Amsterdam, The Netherlands.

*Corresponding author: Jan R.T. van Weering, Center for Neurogenomics and Cognitive Research, VU University, De Boelelaan 1085, 1081 HV Amsterdam, The Netherlands. Email: jan.van.weering@vu.nl

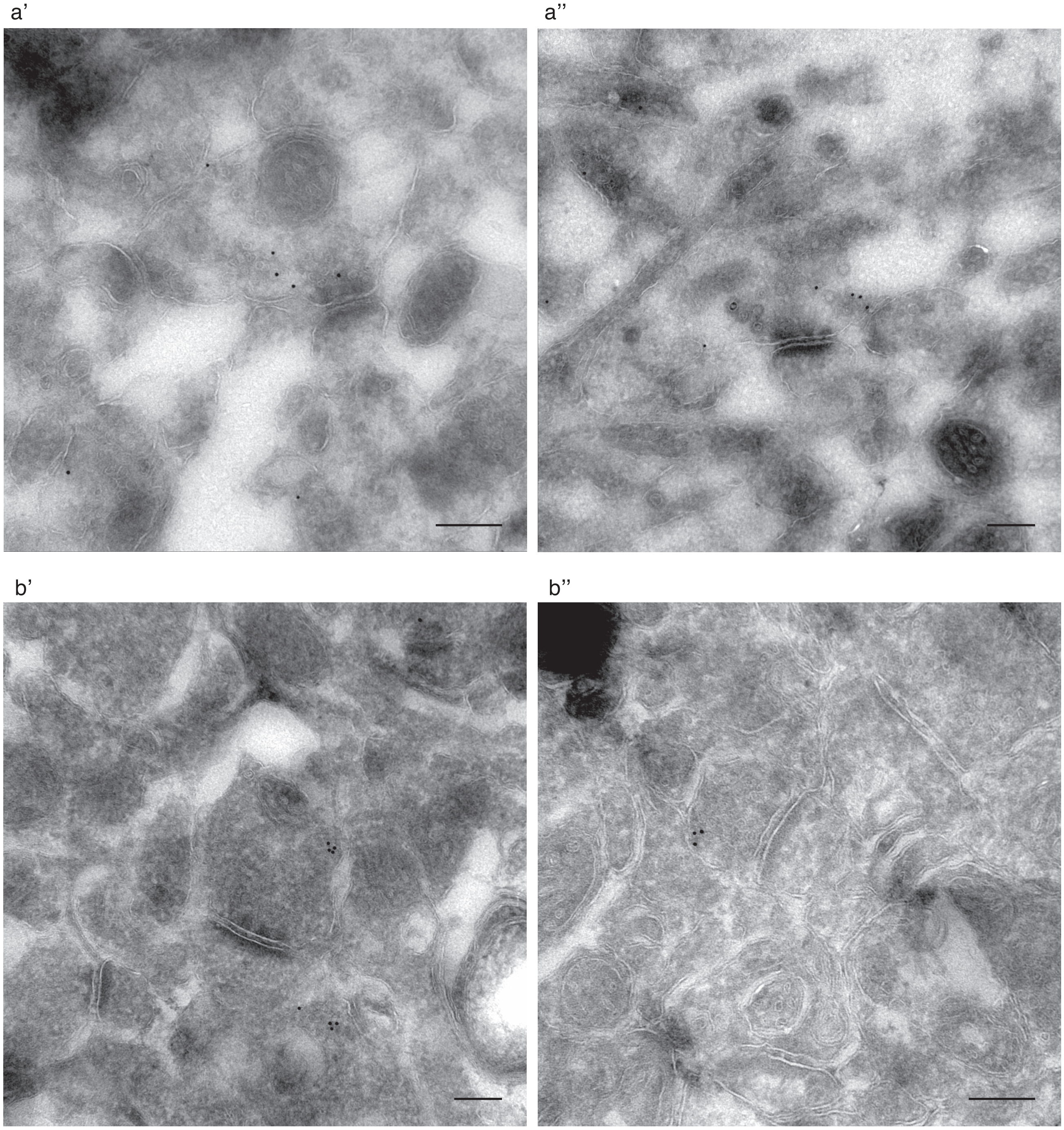

Supplementary Figure S1: Uncropped electron micrographs of the presynaptic localization of VPS35. (**a'** and **a''**) Corresponding images from Figure 1 e' and e'' using the rabbit antibody against VPS35. N=2 animals. (**b'** and **b''**) Corresponding images from Figure 1 f' and f'' using the goat antibody against VPS35. N=2 animals. Scale bar=200 nm.

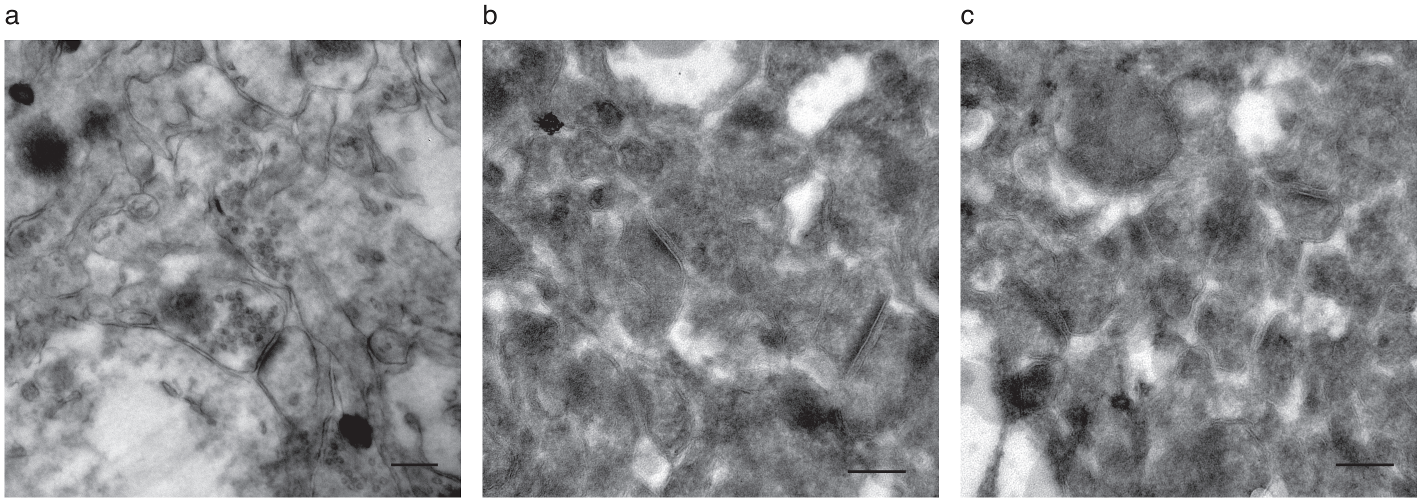

Supplementary Figure S2: Electron micrographs of the negative controls for immunoelectron microscopy against VPS35. (**a**) Negative control processed in parallel with the immunolabelling with VPS35 Cat. No. ab10099, but preincubating the primary antibody with the blocking peptide Cat. No. ab23181 at a ratio 5:1, Figure 1c-d. Scale bar=250nm. (**b** and **c**) Negative control processed in parallel with the immuno-gold labelling with VPS35, but without adding primary antibody, just bridging antibody and Protein A-gold, Figure 1e-f. Scale bar=200nm.

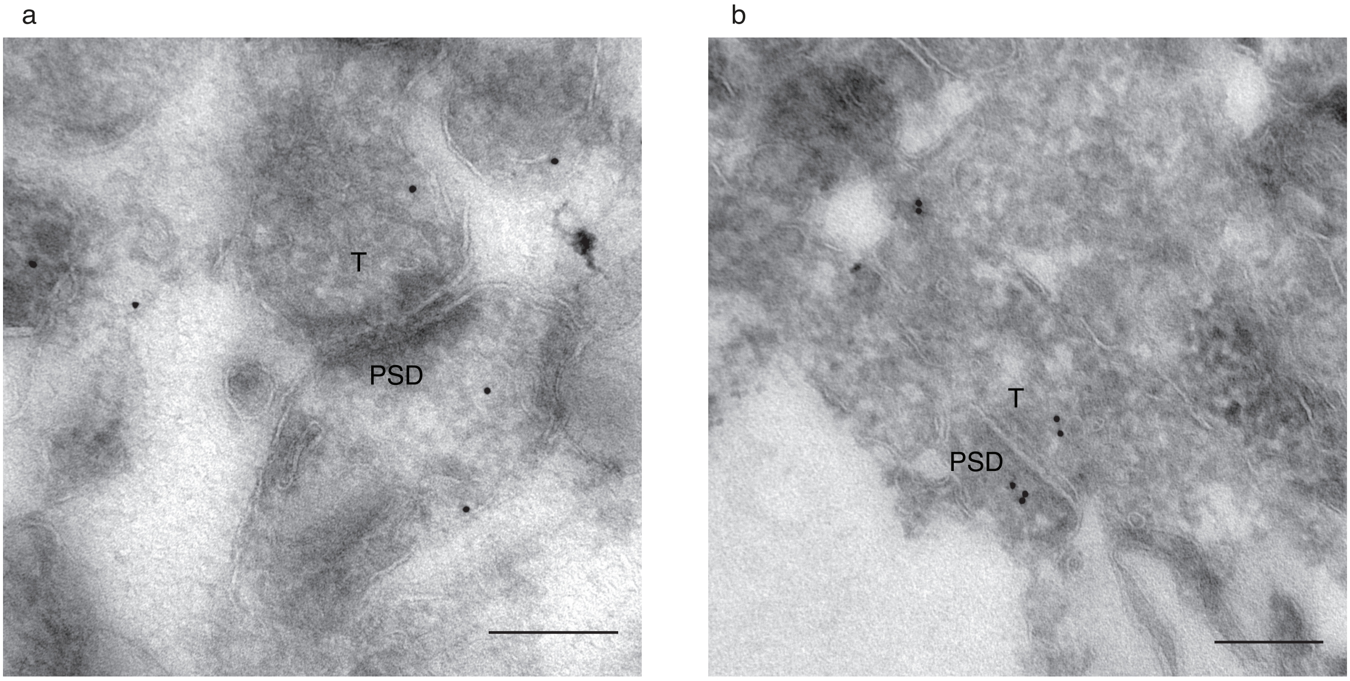

Supplementary Figure S3: Electron micrographs of the postsynaptic localization of VPS35. (**a**) Using the rabbit antibody against VPS35. N=2 animals. (**b**) Using the goat antibody against VPS35. N=2 animals. Scale bar=200 nm. 'PSD' indicates postsynaptic density and 'T' the presynaptic terminal.

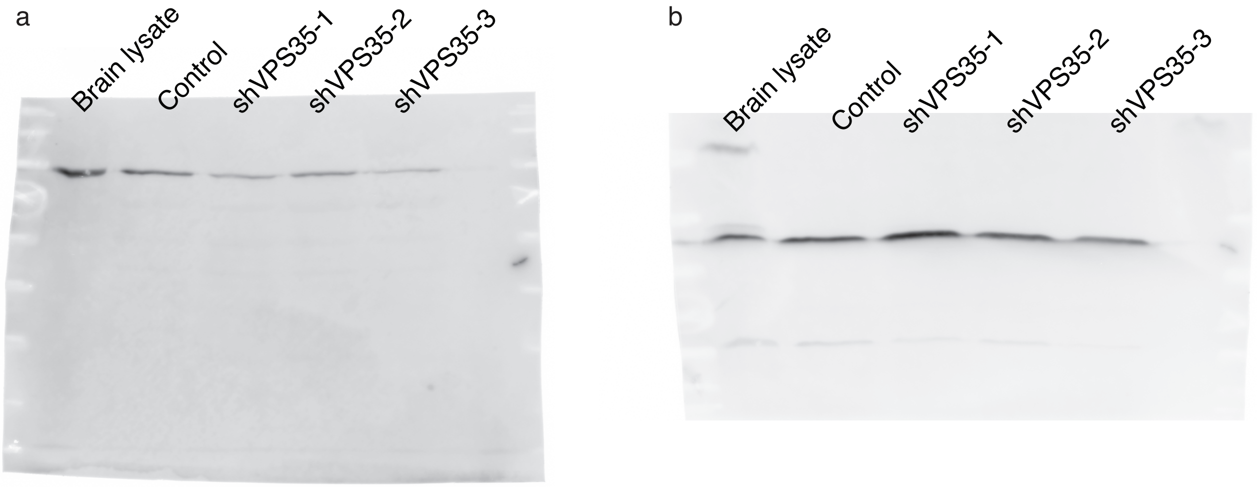

Supplementary Figure S4: Original uncropped blots for (a) VPS35 and (b) actin of the data shown in Figure 2b. The brain lysate condition serves as a technical control and is left out in Figure 2.

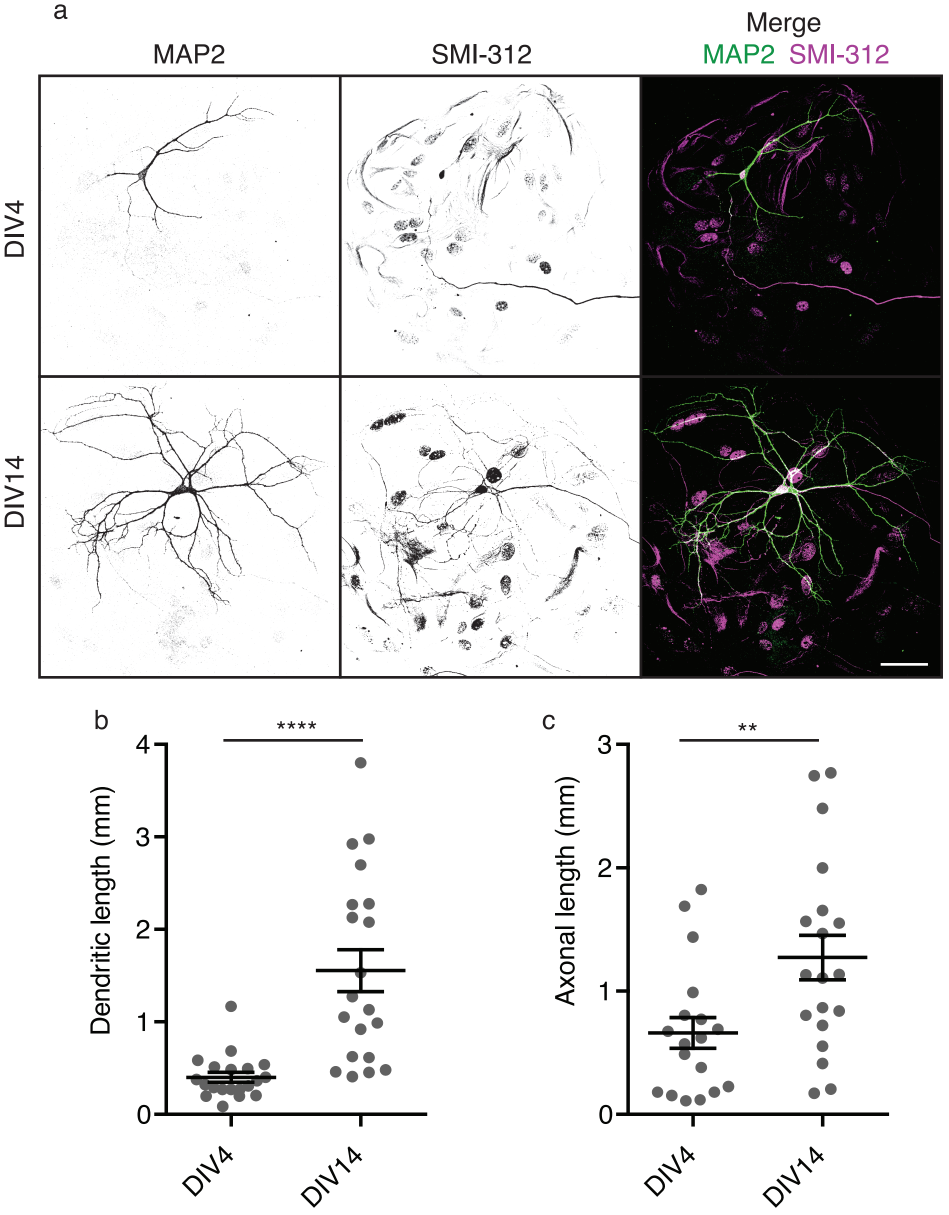

Supplementary Figure S5. Increase in dendritic and axonal length during development is detected using SynD. (**a**) Representative confocal microscopy images of hippocampal autaptic wild type neurons at DIV4 and DIV14 stained with MAP2 and SMI-312. Scale bar=50 µm (**b**) Quantification of the dendritic length (n=20 neurons, N=2 animals). (**c**) Quantification of the axonal length (n=20 neurons, N=2 animals). Detailed information (average, SEM, n and statistics) is available in in Table S1.

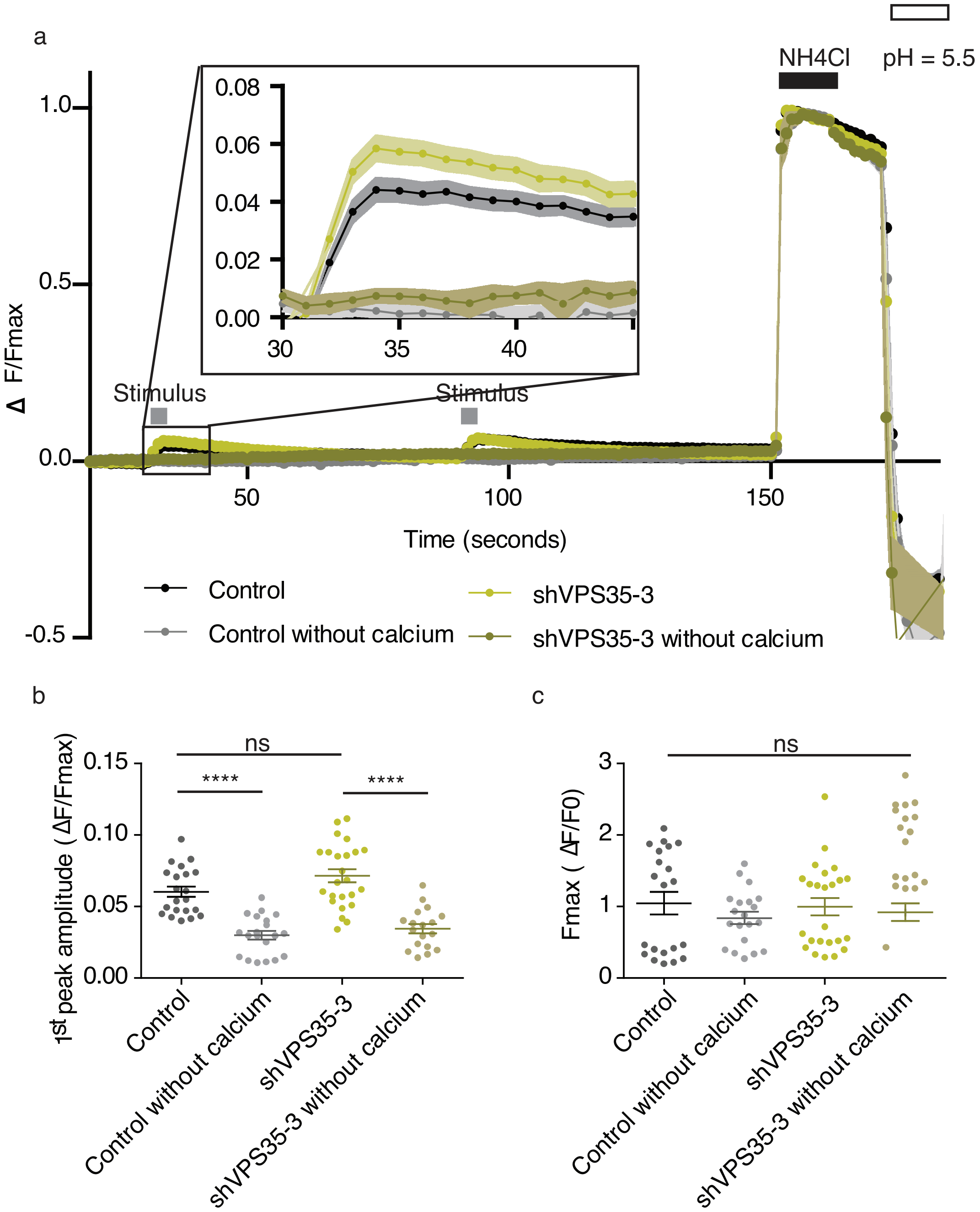

Supplementary Figure S6: SynaptopHluorin reports the absence of calcium in the extracellular medium. (**a**) Time course of synaptopHluorin fluorescence during the imaging protocol, plotted as Δ F/Fmax. The grey boxes indicate the electrical stimulation (100 AP, 40 Hz, 30 mV each one), the black box the duration of the exposure to NH4Cl and the white box the duration of the exposure to pH=5.5. (n=21±3 fields of view, N=2 animals). (**b**) Maximum response amplitude during the electrical stimulation plotted as ΔF/Fmax. (**c**) Maximum synaptopHluorin levels during exposure to NH4Cl. Detailed information (average, SEM, n and statistics) is available in in Table S1.

Supplementary Table S1: Summery of the mean, SEM, n/N numbers and statistic reports of all measured variables in the study. Independent field of view (n), independent animal (N), not applicable (empty cells).

Figure	measured variable	Group	Mean ± SEM	n/N	statistics	p-value
1b	Mander's coefficient	VAMP2 in VPS35	0.22 ± 0.01	78/3		
		VPS35 in VAMP2	0.35 ± 0.02	78/3		
2c	Relative VPS35 levels (ICC)	Control	1 ± 0.049	40/3	H = 126.5, p<0.0001	
		shVPS35-1	0.18 ± < 0.01	40/3		<0.0001
		shVPS35-2	0.68 ± 0.03	41/3		0.0524
		shVPS35-3	0.19 ± 0.01	42/3		<0.0001
2d	Relative VPS35 levels (WB)	Control	1 ± 0.14	7	H = 16.1, p=0.0011	
		shVPS35-1	0.25 ± 0.04	7		0.0053
		shVPS35-2	0.58 ± 0.11	7		0.4866
		shVPS35-3	0.20 ± 0.06	5		0.0014
2f	Relative GluA1 levels (ICC)	Control	1 ± 0.34	35/3	H = 33.06, p<0.0001	
		shVPS35-1	0.64 ± 0.15	35/3		< 0.0001
		shVPS35-2	0.72 ± 0.31	36/3		0.0029
		shVPS35-3	0.58 ± 0.28	35/3		< 0.0001
3c	Dendritic length (μm)	Control	2122 ± 120	56/5	H = 7.61, p=0.0547	
		shVPS35-1	1750 ± 89	57/5		
		shVPS35-2	1747 ± 92	64/5		
		shVPS35-3	1775 ± 139	48/5		
3d	Axonal length (μm)	Control	2952 ± 328	26/3	H = 0.23, p=0.9709	
		shVPS35-1	3142 ± 335	27/3		
		shVPS35-2	3200 ± 330	33/3		
		shVPS35-3	3022 ± 394	18/3		
3f	Synapses/μm (VAMP2)	Control	0.19 ± < 0.01	41/3	H = 19.35, p=0.0002	
		shVPS35-1	0.20 ± 0.01	29/3		>0.9999
		shVPS35-2	0.15 ± < 0.01	31/3		0.0011
		shVPS35-3	0.16 ± < 0.01	28/3		0.0127
3g	VAMP2 (a.u.)	Control	74.06 ± 5.16	41/3	H = 2.60, p=0.4562	
		shVPS35-1	77.09 ± 6.59	29/3		
		shVPS35-2	69.47 ± 5.25	31/3		
		shVPS35-3	80.64 ± 5.40	28/3		

Figure	measured variable	Group	Mean ± SEM	n/N	statistics	p-value
3h	Synaptophysin-1 (a.u)	Control	82.58 ± 6.34	41/3	H = 2.30, p=0.5113	
		shVPS35-1	76.19 ± 8.24	29/3		
		shVPS35-2	77.97 ± 6.57	31/3		
		shVPS35-3	90.52 ± 6.63	28/3		
3i	Synapses/μm (bassoon)	Control	0.29 ± 0.01	18/2	H = 8.02, p=0.0454	
		shVPS35-1	0.25 ± 0.01	18/2		
		shVPS35-2	0.27 ± 0.01	19/2		
		shVPS35-3	0.29 ± 0.01	18/2		
4b	Active zone length (μm)	Control	0.54 ± 0.02	154/3	H = 7.81, p=0.0501	
		shVPS35-1	0.49 ± 0.02	161/3		
		shVPS35-2	0.48 ± 0.01	163/3		
		shVPS35-3	0.47 ± 0.01	160/3		
4c	# Synaptic vesicles/synapse	Control	90.14 ± 5.51	159/3	H = 6.06, p=0.1085	
		shVPS35-1	99.13 ± 7.03	164/3		
		shVPS35-2	88.10 ± 4.70	165/3		
		shVPS35-3	105.40 ± 6.02	162/3		
4d	# docked synaptic vesicles/synapse	Control	4.91 ± 0.22	159/3	H = 0.03, p=9980	
		shVPS35-1	4.95 ± 0.21	164/3		
		shVPS35-2	4.91 ± 0.20	165/3		
		shVPS35-3	4.76 ± 0.17	162/3		
5c	1st peak amplitude (ΔF/Fmax)	Control	0.14 ± < 0.01	31/4	H =6.74, p = 0.0343	
		shVPS35-2	0.19 ± 0.01	19/4		0.0211
		shVPS35-3	0.16 ± 0.01	14/4		> 0.9999
5d	% Active synapses	Control	93.89 ± 1.09	31/4	H =0.05, p = 0.9751	
		shVPS35-2	91.11 ± 2.55	19/4		
		shVPS35-3	91.77 ± 2.52	14/4		
5e	Fmax (a.u.)	Control	1584 ± 28	31/4	H =13.33, p = 0.0013	
		shVPS35-2	1442 ± 28	19/4		0.0014
		shVPS35-3	1469 ± 29	14/4		0.0277
6c	1st peak amplitude (ΔF/Fmax)	Control	0.15 ± <0.01	26/3	F(3,92) =1.5227, p=0.2127	
		shVPS35-1	0.13 ± < 0.01	22/3		
		shVPS35-2	0.13 ± <0.01	23/3		
		shVPS35-3	0.13 ± <0.01	25/3		
6d	% Active synapses	Control	89.03 ± 2.08	26/3	H = 13.32, p=0.0040	
		shVPS35-1	70.50 ± 4.58	22/3		0.0012
		shVPS35-2	81.71 ± 3.62	23/3		0.3297
		shVPS35-3	87.11 ± 1.80	25/3		> 0.9999

Figure	measured variable	Group	Mean ± SEM	n/N	statistics	p-value
6e	Fmax (a.u.)	Control	5726 ± 487	26/3	H = 14.25, p=0.0026	
		shVPS35-1	3711 ± 239	22/3		0.0034
		shVPS35-2	4206 ± 258	23/3		0.1957
		shVPS35-3	5490 ± 491	25/3		> 0.9999
6f	F Baseline (a.u)	Control	2841 ± 117	26/3	H = 8.81, p=0.0318	
		shVPS35-1	2251 ± 132	22/3		0.0226
		shVPS35-2	2355 ± 66	23/3		0.4927
		shVPS35-3	2863 ± 198	25/3		> 0.9999
6g	Ratio peak amplitude (2nd/1st)	Control	1.21 ± 0.03	26/3	H = 2.26, p=0.4532	
		shVPS35-1	1.08 ± 0.05	22/3		
		shVPS35-2	1.21 ± 0.03	23/3		
		shVPS35-3	1.15 ± 0.04	25/3		
6h	F pH = 5.5 (a.u.)	Control	1881 ± 214	26/3	H = 6.56, p=0.0870	
		shVPS35-1	1621 ± 150	22/3		
		shVPS35-2	1883 ± 173	23/3		
		shVPS35-3	1787 ± 120	25/3		
S4b	Dendritic length (mm)	DIV4	0.40 ± 0.05	20/2	U = 36.00, p<0.0001	
		DIV14	1.55 ± 0.22	20/2		
S4c	Axonal length (mm)	DIV4	0.66 0 ± 0.12	20/2	U = 85.00, p=0.0082	
		DIV14	1.27 ± 0.18	20/2		
S5b	1st peak amplitude (ΔF/Fmax)	Control	0.06 ± <0.01	21/2	H = 46.45, p<0.0001 (shVPS35-3 vs shVPS35-3 without calcium p<0.0001)	
		Control without calcium	0.02 ± < 0.01	20/2		<0.0001
		shVPS35-3	0.06 ± <0.01	24/2		> 0.9999
		shVPS35-3 without calcium	0.02 ± <0.01	18/2		
S5b	Fmax (ΔF/F0)	Control	1.04 ± 0.15	21/2	H = 0.89, p=0.8256	
		Control without calcium	0.83 ± 0.08	20/2		
		shVPS35-3	0.99 ± 0.12	24/2		
		shVPS35-3 without calcium	0.92 ± 0.12	18/2		
